# Commensal *Pseudomonas* Species Isolated from Wastewater and Freshwater Milieus in the Eastern Cape Province, South Africa, as Reservoir of Antibiotic Resistant Determinants

**DOI:** 10.3390/ijerph9072537

**Published:** 2012-07-23

**Authors:** Isoken H. Igbinosa, Uchechukwu U. Nwodo, Anibal Sosa, Mvuyo Tom, Anthony I. Okoh

**Affiliations:** 1 Applied and Environmental Microbiology Research Group (AEMREG), Department of Biochemistry and Microbiology, University of Fort Hare, Private Bag X1314, Alice 5700, South Africa; Email: ladyisk@yahoo.com (I.H.I.); uchenwodo@gmail.com (U.U.N.); mtom@ufh.ac.za (M.T.); 2 Former Director, International Program & Clinical Advisor, Alliance for the Prudent Use of Antibiotics (APUA), 75 Kneeland Street, Boston, MA 02111, USA; Email: adejsosa@massmed.org

**Keywords:** *Pseudomonas*, commensal, multidrug resistant genes, integron, *bla_TEM_*

## Abstract

*Pseudomonas* species are opportunistic pathogens with implications in a wide range of diseases including cystic fibrosis and sickle cell anaemia. Because of their status as multidrug resistant (MDR) and extremely drug resistant (XDR) bacteria *Pseudomonas* species represent a threat to public health. Prevalence, antibiogram and associated antibiotic resistant genes of *Pseudomonas* species isolated from freshwater and mixed liquor environments in the Eastern Cape Province of South Africa were assessed. Polymerase chain reaction (PCR) based technique was used to identify the isolates and screen for antibiotic resistant genes. The result shows occurrence of *Pseudomonas* spp. in freshwater and mixed liquor as follows: 71.42% and 37.5% (*P. putida*), 14.28% and 31.25% (*P. flourescens*), 7.14% and 6.25% (*P. aeruginosa*) and 7.14% and 25% for other *Pseudomonas* species respectively. Disk diffusion antibiogram of the *Pseudomonas* isolates from the two locations showed 100% resistance to penicillin, oxacillin, clindamycin, rifampicin and 100% susceptibility to ciprofloxacin and gentamicin with varied percentage resistances to cephalothin, nalidixic acid, tetracycline, and ampicillin. The *bla_TEM_* antibiotic resistant gene was detected in 12.5% of *P. putida*, 57.14% of *P. fluorescens*, 100% *P. aeruginosa* and 40% in other *Pseudomonas* species. Similarly, Integrons conserved segment were detected in 12.5% of *P. putida*, 57.14% of *P. fluorescens*, 100% of *P. aeruginosa* and 40% of other *Pseudomonas* species. The presence of *bla_TEM_* gene and integrons conserved segment in some of the isolates is worrisome and suggest *Pseudomona*s species as important reservoirs of multidrug resistance genes in the Eastern Cape Province environment.

## 1. Introduction

Antibiotic resistance by bacteria has been recognized as a major medical problem facing humankind and to prevent this scourge, the knowledge of their antibiotic susceptibilities, antibiotic resistance genes and their dissemination is required [1]. Most studies on antibiotic resistance in the environment have focused on enteric pathogens including *Escherichia coli* [2], Enterococci [3], *Aeromonas* spp. [4] and *Campylobacter* [5]. However, antibiotic resistant bacteria in the environments are autochthonous, and as reservoirs of antibiotic resistant determinants, they could perpetuate the spread of antibiotic-resistance genes to human and animal pathogens by horizontal gene transfer through such mobile genetic elements as plasmids, transposons and integrons [2], especially in wastewater treatment facilities (WWTP) [6,7]. Integrons are genetic elements that aid the acquisition and expression of gene cassettes in bacteria, most of them are involved in antibiotic resistance. WWTP have been reported as important reservoirs of antibiotic resistant organisms/determinants which could persist in the treated effluent and subsequently released into the natural environment [8,9,10] and thus impact on the ecology of antimicrobial resistance in bacterial populations [11,12,13]. However, reports of commensal bacteria including the pseudomonads as sources of antibiotic resistance determinants in the environment are rare. 

*Pseudomonas* species are Gram negative motile rods belonging to the family Pseudomonaceae and found in various environments. Their ability to utilize different organic compounds as carbon and energy source as well as survival in the apparent absence of nutrients has been attributed to their genetic versatility which translates into enhanced metabolic activity with exceptional ability to adapt and colonize a wide variety of ecological niches including water, soil and rhizosphere [14]. *Pseudomonas* spp. are so well adapted in their environment that they survive extremes which includes temperatures ranging from 4 °C to 43 °C, and weak ion concentrations, among others. In this study, we assessed the incidence of *Pseudomonas* spp. in some freshwater environment and wastewater in the Eastern Cape Province of South Africa as well as the prevalence of antibiotic resistance genes in the isolates.

## 2. Materials and Methods

### 2.1. Sample Collection

The freshwater samples were collected from Kat river is situated in Fort Beaufort (geographical coordinates: S 32° 47.071′ E 026° 38.916′) and Tyume river in Alice (geographical coordinates: S 32° 46.629′ E026° 50.149′) in the Eastern Cape Province, South Africa. Similarly, the mixed liquor samples were collected from two wastewater treatment plants located in Fort Beaufort and Alice. The plants are relatively small with design capacities of 2–3 ML/day and operate using activated sludge technology. While the Alice plant empties its final effluent into the Tyume River, the Fort Beaufort plant empties its effluents into the Kat River. The latest Green Drop report on both plants suggests that they are deserving of attention towards ensuring that they produce effluents of acceptable qualities [15]. These samples were transported in cooler boxes to the laboratory of the Applied and Environmental Microbiology Research Group (AEMREG) University of Fort Hare, Alice for microbiological analyses. Sampling was conducted once during the four seasons of the year (autumn, winter spring, and summer). 

### 2.2. Isolation Processing of Samples

All freshwater and wastewater samples were serially diluted and 100 µL of the diluted samples were plated on Glutamate Starch Phenol-red (GSP) agar and incubated overnight at 37 °C. Pseudomonas-like isolates were counted, isolated and purified on fresh GSP agar. Purified isolates were thereafter transferred unto Nutrient agar plates and incubated overnight at 37 °C and thereafter screened based on typical morphology, catalase and oxidase reactions. 

### 2.3. Identification of Isolates by Polymerase Chain Reaction (PCR)

The purified isolates were grown on Nutrient agar for 24 h, and afterwards cells were harvested into 100 µL nuclease free water in 1.5 mL eppendorf tubes and homogenized by vortexing. The tubes were then placed in a heating block (Dri-block DB.2A, Techne, SA) at 100 °C for 10 min. After heating, the tubes were centrifuged at 25 °C for 3 min at 11,000 rpm (revolutions per minute) and immediately placed on ice. The supernatant was transferred to a new tube and used directly as DNA template for PCR assay [16]. Specific primers for *Pseudomonas* genus; PA-GS-F (5′-GACGGGTGAGTAATGCCTA-3′), and PA-GS-R (5′-CACTGGTGTTCCTTCCTATA-3′) were used in a 50 µL PCR reaction [17]. PCR conditions were as follows: 95 °C for 5 min, 10 cycles of 94 °C for 15 s, 53 °C for 30 s and 72 °C for 45 s; this was repeated for another 25 cycles with the exception of the 72 °C elongation step, which was increased by 5 seconds for every cycle; a final extension phase of 72 °C for 10 min was used. *Pseudomonas aeruginosa* reference strain ATCC 27853 was used as positive control and a reaction mixture containing Nuclease free water as negative control. The amplified PCR products of 617 bp were analysed by gel electrophoresis in 0.8% agarose gels stained with ethidium bromide (EtBr) 0.5 mg/L, for 1 h at 100 V in 0.5 × TAE buffer (40 mM Tris-HCl, 20 mM Na-acetate, 1 mM EDTA, pH 8.5) and then visualized and photographed with a imaging system Alliance 4.7 XD-79 (UVITEC Cambridge).

### 2.4. Specie Specificity Screening of *Pseudomonas isolates*

All isolates confirmed to belong to the *Pseudomonas* genus were further screened for three specific species of interest (*P. fluorescens*, *P. aeruginosa* and *P. putida*) selected based on on the dominance of these species from the results obtained on the preliminary identification carried out using API 20NE kit (data not shown) using the sets of primers listed in Table 1. The PCR conditions were as follows: *P. fluorescens* (2 min at 94 °C; 5 cycles consisting of 94 °C for 45 s, 55 °C for 1 min, 72 °C for 2 min; 35 cycles consisting of 92 °C for 45 s, 60 °C for 45 s, 72 °C for 2 min; final extension of 72 °C for 2 min; and final cooling at 4 °C); *P. aeruginosa* (95 °C for 1 min; 40 cycles of denaturation at 95 °C for 15 s, annealing at 58 °C for 20 s; final extension at 68 °C for 40 s); *P. putida* (initial denaturation at 95 °C for 10 min, then 30 cycles of denaturation at 94 °C for 30 s, annealing at 55 °C for 90 s and extension at 72 °C for 7 min).

**Table 1:** List of Primers used in this study.

Target genes	Sequences 5'–3'	Amplicon size (bp)	References
*P. aeruginosa*	GGCGTGGGTGTGGAAGTC	199	[18]
	TGGTGGCGATCTTGAACTTCTT		
*P. putida*	TCACCTCCGAGGAAACCAGCTTG	676	[19]
	TCTGTTGTGAACGCCCTGTC		
*P. fluorescens*	TGCATTCAAAACTGACTG	850	[20]
	AATCACACCGTGGTAACCG		
*bla_TEM_* gene	AGGAAGAGTATGATTCAACA	535	[21]
	CTCGTCGTTTGGTATGGC		
*TetC* gene	GGTTGAAGGCTCTCAAGGGC	505	[22]
	GGTTGAAGGCTCTCAAGGGC		
Integrons conserved segment	GGCATCCAAGCAGCAAG	Variable	[23]
	AAGCAGACTTGACCTGA		
*bla_OXA_* gene	TGAGCACCATAAGGCAACCA	311	[24]
	TTGGGCTAAATGGAAGCGTTT		
*bla_amp_C*	GGTATGGCTGTGGGTGTTA	882	[25]
	TCCGAAACGGTTAGTTGAG		

### 2.5. Antibiotic Susceptibility Testing

Antimicrobial susceptibility testing was performed using the disk diffusion method [17] with Muller-Hinton agar as the growth medium. Antibiotics were selected to represent some major classes of antibiotic and anti-pseudomonal antibiotics used as first line drug for pseudomonal infections. Antibiotics used in the study include penicillins (10 µg), clinamycins (2 µg), ciprofloxacin (5 µg), rafamycin (5 µg), trimethoprim (5 µg), sulphamethoxazole (25 µg), gentamicin (10 µg), chloramphenicol (30 µg), tetracycline (10 µg), erythromycin (15 µg), minocycline (30 µg), vacomycin (30 µg), cefotaxime (30 µg), nalidixic acid (30 µg), nitrofurantoin (300 µg), cephalothin (30 µg), ofloxacin (5 µg), ampicillin (25 µg), ampicillin-sulbactam (20 µg), oxacillin (1 µg). Disks were purchased from Mast Diagnostics (Mast Group, Merseyside, UK). *Pseudomonas* isolates were identified as susceptible, intermediate or resistant according to the National Committee for Clinical Laboratory Standard Guidelines (CLSI) [26].

### 2.6. PCR Detection of Antibiotic Resistant Genes

The DNA of the *Pseudomonas* isolates was extracted following the method of Sambrook and Russell [16]. The set of primers used for the detection of antibiotic resistance genes are shown in Table 1. The PCR reaction was done in a total volume of 25 µL and the following conditions: *bla_TEM_* gene (3 min at 93 °C, 40 cycles of 1 min at 93 °C, 1 min at 55 °C and 1 min at 72 °C and finally 7 min at 72 °C); *bla_OXA_* gene and *bla_amp_C* gene (94 °C for 5 min, 30 cycles of 25 s of denaturation at 94 °C, 40 s of annealing at 53 °C and 50 s of extension at 72 °C and a final cycle at 7 min at 72 °C); *TetC* gene (3 min at 94 °C, followed by 30 cycles of 1 min at 94 °C, 1 min at 65 °C and 1 min at 72 °C followed by 10 min at 72 °C); Integrons conserved segment (initial denaturation at 94 °C for 12 min, 1 min of denaturation at 94 °C, 1 min of annealing at 55 °C and 5 min of extension at 72 °C for a total of 35 cycles; five seconds were added to the extension time at each cycle).

## 3. Results

### 3.1. Molecular Identification of Isolates

Molecular identification of the *Pseudomonas* at both genus and specie levels were carried out using the sets of primers shown in Table 1. Sixty isolates were identified to belong to the *Pseudomonas* genus, twenty eight (46.7%) of which were from freshwater, and 32 (53.3%) were from the wastewater mixed liquor. Freshwater and wastewater samples collected in the four seasons (winter, autumn, spring and summer) showed incidences of *Pseudomonas* of 50% (autumn), 21.43% (spring) and 28.57% (summer) respectively in freshwater samples with none detected in winter (Table 2). Similarly, distribution of *Pseudomonas* with respect to species were 85.71% for *P. putida* (autumn) and 14.29% for other *Pseudomonas* species. In spring, the distribution was 33.33% each for *P. putida*, *P*. *aeruginosa* and *P. fluorescens* (Table 2). Furthermore, the analysis of the mixed liquor showed incidences of *Pseudomonas* at 81.25% (spring) and 18.75% (summer) alone, but at the species level, the following were observed; 66.67% (*P. putida*) and 33.33% (*P. fluorescens*) during summer, and 30.77% each for *P. putida*, *P. flourescens* and other *Pseudomonas* spp. and 7.69% for *P. aeruginosa* during spring (Table 3), respectively.

**Table 2:** Prevalence of *Pseudomonas* species in freshwater samples.

Seasons^ a^	*P. aeruginosa*		*P. putida*		*P. flourescens*		Other *Pseudomonas* spp.	
	Alice (%)	FBF (%)	Alice (%)	FBF (%)	Alice (%)	FBF (%)	Alice (%)	FBF (%)
Autumn	0	0	12 (85.71)	0	0	0	2 (14.29)	0
Winter	0	0	0	0	0	0	0	0
Spring	2 (33.33)	0	2 (33.33)	0	2 (33.33)	0	0	0
Summer	0	0	0	6 (75)	0	2(25)	0	0

^a^ Summer (November to March); autumn (April to May); winter (June to August); spring (September to October). Alice and Fort Beaufort (FBF) represents sampling locations.

**Table 3:** Prevalence of *Pseudomonas* species in mixed liquor samples.

Seasons^ a^	*P. aeruginosa*		*P. putida*		*P. fluorescens*		Other *Pseudomonas* spp.	
	Alice (%)	FBF (%)	Alice (%)	FBF (%)	Alice (%)	FBF (%)	Alice (%)	FBF (%)
Autumn	0	0	0	0	0	0	0	0
Winter	0	0	0	0	0	0	0	0
Spring	0	2 (7.69)	6 (23.08)	2 (7.69)	6 (23.08)	2 (7.69)	2 (7.69)	6 (23.08)
Summer	0	0	0	4 (66.67)	0	2 (33.33)	0	0

^a^ Summer (November to March); autumn (April to May); winter (June to August); spring (September to October). Alice and Fort Beaufort (FBF) represents sampling locations.

In general, with respect to the freshwater samples collected from Alice, 70% of the isolates recovered were *P. putida*, while the remaining 30% were equally (10% each) made up of *P. fluorescens*, *P. aeruginosa* and other *Pseudomonas* spp. For the Fort Beaufort water samples, *P. putida* constituted 75% of the isolates, while the remaining 25% were *P. fluorescens*. With respect to the mixed liquor samples from Alice, *P. putida*, and *P. fluorescens* made up 42.85% each of the isolates and the other *Pseudomonas* spp. constituted 14.29%. For the Fort Beaufort mixed liquor samples, the isolates composition includes 33.33% (*P. putida*), 22.22% (*P. fluorescens*)*,* 11.11% (*P. aeruginosa*) and 33.33% (other *Pseudomonas* spp.). 

### 3.2. Antibiotic Susceptibility Profile

The antibiograms of the *Pseudomonas* species are as shown in Table 4 and Table 5. All isolates (100%) from the two sites were susceptible to ciprofloxacin and gentamicin. Conversely, all (100%) were resistant to penicillin, oxacillin, vacomycin, trimethoprim, clindamycin and rifampicin. Varied resistances were observed against nitrofurantion as isolates from Alice showed the following resistance regimes; 60% (*P. putida*), 50% (*P. fluorescens*), 100% (*P. aeruginosa*) and 50% against other *Pseudomonas* spp. (Table 4). Unlike isolates from Alice, those from Fort Beaufort showed 100% resistance to nitrofurantoin. Isolates from Alice showed varied susceptibilities to cefotaxime in the order 60% (*P. putida*), 50% (*P. flourescens*), 100% (*P. aeruginosa*) and 50% (other *Pseudomonas* spp.). The antibiotic resistance pattern with respect to cephalothin was 100% each for *P. putida*, *P. aeruginosa* and the other *Pseudomonas* spp., and 75% for *P. fluorescens*. Ampicillin-sulbactam showed activity against *P. putida* (60%) and *P. fluorescens* (75%) (Table 4). Similarly, ofloxacin showed activity against *P. putida* (90%), *P. flourescens* (100%), *P. aeruginosa* (100%) and other *Pseudomonas* spp. (100%). Susceptibilities of isolates from Fort Beaufort to cephalothin were 50% (*P. fluorescens*), while all (100%) the *P. putida, P. aeruginosa* and the other *Pseudomonas* species were resistant (Table 5).

**Table 4:** Antibiotic susceptibilities of *Pseudomonas* species isolated from freshwater and mixed liquor samples from Alice.

Antibiotics		*P. aeruginosa*			*P. putida*			*P. fluorescens*			Other *Pseudomonas* species		
		S (%)	I (%)	R (%)	S (%)	I (%)	R (%)	S (%)	I (%)	R (%)	S (%)	I (%)	R (%)
Penicillin	PG	0	0	100	0	0	100	0	0	100	0	0	100
	AP	0	0	100	40	10	50	25	0	75	0	0	100
	OX	0	0	100	0	0	100	0	0	100	0	0	100
Tetracycline	T	0	100	0	20	40	40	25	25	50	0	50	50
	MN	100	0	0	30	40	30	25	50	25	50	50	0
Quinolones	CIP	100	0	0	100	0	0	100	0	0	100	0	0
	NA	0	100	0	30	40	30	25	25	50	0	50	50
	OFX	100	0	0	90	10	0	100	0	0	100	0	0
Cephems	CTX	100	0	0	60	40	0	50	0	50	50	50	0
	KF	0	0	100	0	0	100	0	25	75	0	0	100
Phenicols	C	100	0	0	20	30	50	25	25	50	50	0	50
Macrolides	E	0	100	0	0	0	100	0	25	75	0	50	50
Aminoglycosides	GM	100	0	0	100	0	0	100	0	0	100	0	0
Glycopeptides	VA	0	0	100	0	0	100	0	0	100	0	0	100
Nitrofurans	NI	100	0	0	20	20	60	25	0	75	50	0	50
Folate pathway inhibitors	TM	0	0	100	30	0	70	0	0	100	0	0	100
	SMX	0	0	100	0	0	100	50	0	50	0	0	100
β-lactams	SAM	0	0	100	60	10	30	75	0	25	0	0	100
Lincosamides	CD	0	0	100	0	0	10	0	0	100	0	0	100
Ansamycins	RP	0	0	100	0	0	100	0	0	100	0	0	100

**Legend:** PG—penicillin, AP—Ampicillin, OX—Oxacillin, T—Tetracycline, MN—Minocycline, CIP—Ciprofloxacin, Na—Nalidixic acid, OFX—Ofloxacin, CTX—Cefotaxime, KF—Cephalothin, C—Chloramphenicol, E—Erythromycin, GM—Gentamicin, VA—Vacomycin, NI—Nitrofurantoin, TM—Trimethoprim, SMX—Sulphamethoxazole, SAM—Ampicillin-sulbactam, CD—Clindamycin, RP—Rifamycin.

**Table 5:** Antibiotic susceptibility of *Pseudomonas* species isolated from freshwater and mixed liquor samples from Fort Beaufort.

Antibiotics		*P. aeruginosa*			*P. putida*			*P. fluorescens*			Other *Pseudomonas* species		
		S (%)	I (%)	R (%)	S (%)	I (%)	R (%)	S (%)	I (%)	R (%)	S (%)	I (%)	R (%)
Penicillin	PG	0	0	100	0	0	100	0	0	100	0	0	100
	AP	0	0	100	0	0	100	0	0	100	33.33	0	66.67
	OX	0	0	100	0	0	100	0	0	100	0	0	100
Tetracycline	T	0	0	100	0	40	60	0	0	100	0	33.33	66.67
	MN	0	0	100	0	80	20	0	50	50	33.33	0	66.67
Quinolones	CIP	100	0	0	100	0	0	100	0	0	100	0	0
	NA	0	0	100	20	0	80	0	100	0	0	33.33	66.67
	OFX	100	0	0	80	0	20	100	0	0	100	0	0
Cephems	CTX	0	0	100	20	60	20	100	0	0	0	33.33	66.67
	KF	0	0	100	0	0	100	50	50	0	0	0	100
Phenicols	C	0	0	100	0	20	80	0	0	100	0	33.33	66.67
Macrolides	E	0	0	100	0	0	100	0	0	100	0	0	100
Aminoglycosides	GM	100	0	0	100	0	0	100	0	0	100	0	0
Glycopeptides	VA	0	0	100	0	0	100	0	0	100	0	0	100
Nitrofurans	NI	0	0	100	0	0	100	0	0	100	0	0	100
Folate pathway inhibitors	TM	0	0	100	0	0	100	0	0	100	0	0	100
	SMX	0	0	100	0	0	100	50	0	50	0	0	100
β-lactams	SAM	0	0	100	0	20	80	100	0	0	33.33	0	66.67
Lincosamides	CD	0	0	100	0	0	100	0	0	100	0	0	100
Ansamycins	RP	0	0	100	0	0	100	0	0	100	0	0	100

**Legend:** PG—penicillin, AP—Ampicillin, OX—Oxacillin, T—Tetracycline, MN—Minocycline, CIP—Ciprofloxacin, Na—Nalidixic acid, OFX—Ofloxacin, CTX—Cefotaxime, KF—Cephalothin, C—Chloramphenicol, E—Erythromycin, GM—Gentamicin, VA—vacomycin, NI—Nitrofurantoin, TM—Trimethoprim, SMX—Sulphamethoxazole, SAM—Ampicillin-sulbactam, CD—Clindamycin, RP—Rifamycin.

### 3.3. Identification of Antibiotic Resistance Genes

The screening for antibiotic resistance genes revealed the absence of *bla_OXA_*, *bla_amp_C* and *TetC* genes as they were not detected in any of the *Pseudomonas* isolates. However, Integron conserved segment was detected in freshwater samples in 10% of *P. putida* and 50% of *P. fluorescens* isolates. On the other hand, integron was found in isolates from mixed liquor in 33.33% (*P. putida*), 80% (*P. flourescens*), 100% (*P. aeruginosa*) and 50% for the other *Pseudomonas* spp. Similarly, *bla_TEM_* gene was detected in these same organisms in the same proportion as Integron conserved segment.

## 4. Discussion

The incidences of *Pseudomonas* species in the studied sampling sites appeared to be season dependent as variation in seasonal distribution reflected different recovery rates of the bacteria. Nevertheless, it must be appreciated that this recovery rates may not represent the total population of viable *Pseudomonas* species present in the samples, but selective for some species based on the incubation temperature used, especially considering that some *Pseudomonas* species such as *P. syringae* do not grow at temperatures above 30 °C. The absence of the *Pseudomonas* during winter suggests that the recovered isolates could not strive at low temperature in line with their mesophilic nature. Higher prevalence of *Pseudomonas* isolates were recovered during spring followed by summer especially in the mixed liquor samples, suggesting that warmer temperature favoured the recovery of these isolates. Freshwater samples from Alice consistently showed higher incidences of *Pseudomonas* species when compared to Fort Beaufort as evident from the incidence of 71.42% compared to that of Fort Beaufort (28.57%). Conversely, a relatively higher number of isolates was recovered in mixed liquor from Fort Beaufort (56.25%) as against Alice (43.25%). These variations may be attributed to human activities at various sites of the rivers, however; this explanation will not suffice for mixed liquor, although the limitations on overreliance on one primer pair/species for speciation of the Pseudomonas species must be appreciated. Similarly, the variation of incidence with season needs to be further investigated as it is not clear why season play a role in the occurrence of *Pseudomonas* species. 

Resistance to different classes of antibiotics shown by the *Pseudomonas* species isolated from both freshwater and mixed liquor is an indication of the potential of the environment as a reservoir for antibiotic resistant organisms. Wastewater treatment process has been put forward as a potential vehicle for the selective enhancement and increase of multidrug resistant bacteria in the aquatic environment [8]. Although the findings of Gilliver *et al*. [27] in England reported on the occurrence of acquire antibiotic resistance characters in faecal bacteria from wild rodents in woodland sites and Pallecchi *et al*. [28] who studied a secluded population of the Peruvian Amazonas observed the presence of *qnrB* gene in commensal enterobacteria, both findings were with no prior exposure to antibiotics, as the areas were remote and devoid of human activities. Nevertheless the report by Osterblad *et al*. [29] and Thaller *et al*. [30] potentiate the presence of anthropic activities as a selective enrichment of multidrug resistant bacteria in the environment which is in accordance with the results of our findings, and suggests that the restriction of the misuse and overuse of antibiotics is a vital instrument in antibiotic resistance control. 

The antibiotics, ciprofloxacin and gentamicin are the only broad spectrum antibiotics that showed high activities against all the isolates, while other antibiotics showed little or no activity against them, thus suggesting these isolates to be multi-drug resistant. However, an intriguing situation arose where all isolates were resistant to oxacillin but *bla*_OXA_ gene was not detected in any of the isolates, despite that *bla*_OXA_ codes for oxacillin resistance. Hence, it becomes obvious that resistance to antibiotics may be a function of more than one gene, or better still a combination of both genetic and environmental factors.

Li *et al*. [7] reported the presence of *bla_TEM_* in 17.3% of the bacteria isolated from penicillin production wastewater treatment plant effluent and 11% from the river downstream the plant, however, in these organisms, *bla_OXA_* gene was not detected. Similar trend was observed in our current study, however, *bla_TEM_* gene was detected only in isolates from mixed liquor, portending mixed liquor as a reservoir for antibiotic resistant genes. Also, noting that *Pseudomonas* is the most competent bacteria with regards to DNA uptake [31] and ability to produce transformants [32] in different environmental conditions, the chances that it picks up genes from the environment is high and this could very well be the source of these resistance genes in the isolates. The *Pseudomonas* isolates from freshwater and mixed liquor showed the presence of integrons, however those from mixed liquor similarly harbour *bla_TEM_* gene. The survival of *bla_TEM_* gene containing *Pseudomonas* species in wastewater treatment processes [33] could result in the dissemination of the β-lactamase genes into the environment and consequently increase the risk of the environment as reservoirs of antibiotic resistance determinants. 

## 5. Conclusions

*Pseudomonas* species pose a threat to public health as it has been shown to harbour some antibiotic resistance genes. These resistance genes could be transferred to pathogenic organisms, and result in difficulty in treatment and limitation in treatment options. Although resistance is shown to various antibiotics; they have high susceptibilities to ciprofloxacin and gentamicin. Similarly, the presence of antibiotic resistance genes in these environmental isolates suggests *Pseudomonas* species as carriers and sources of antibiotics resistant genes with the potential to disseminate these genes into the environment for other organisms to pick up or transfer horizontally to other competent bacteria. A detailed assessment of the role of season on the incidence of *Pseudomonas* species, and how their antibiotic resistant genes contribute individually and collectively to antibiotic resistance is a subject of ongoing investigation in our group.
